# Operative Treatment of Terrible Triad of the Elbow via Posterolateral and Anteromedial Approaches

**DOI:** 10.1371/journal.pone.0124821

**Published:** 2015-04-24

**Authors:** Hong-wei Chen, Guo-dong Liu, Shan Ou, Jun Fei, Gang-sheng Zhao, Li-jun Wu, Jun Pan

**Affiliations:** 1 Department of Orthopedics, Central Hospital of Yiwu City, Yiwu 322000, China; 2 8th Department, Institute of Surgery Research, Daping Hospital, Third Military Medical University, Chongqing 400042, China; 3 Department of Anesthesiology, General Hospital of Chengdu Military Command, Chengdu 610083, China; 4 Traumatic Center, Research Institute of Surgery, Daping Hospital, Third Military Medical University, Chongqing 400042, China; 5 Digital Medical Research Institute, Wenzhou Medical College, Wenzhou 325027, China; 6 Department of Orthopedics, Second Affiliated Hospital of Wenzhou Medical College, Wenzhou 325027, China; Medical University of South Carolina, UNITED STATES

## Abstract

The aim of the study was to explore the clinical outcome of posterolateral and anteromedial approaches in treatment of terrible triad of the elbow. The study involved 12 patients with closed terrible triad of the elbow treated by posterolateral and anteromedial approaches between January 2010 and June 2012. The mechanism of injury included fall from height in 9 patients and traffic accident in 3. According to O’Driscoll classification for fractures of the ulnar coronoid, there were 11 patients with type Ⅰ and 1 with type Ⅱ fractures. According to Mason classification for fractures of the radial head, there were 3 patients with type Ⅰ, 7 with type Ⅱ and 2 with type Ⅲ fractures. All patients were followed up for 12-27 months (average 15.5 months), which showed no pain or severe pain in all patients except for 2 patients with mild pain. At the last follow-up, the mean flexion was for 125°(range, 90°-140°), the mean extension loss for 20°(range, 0°-70°), the mean pronation for 66°(range, 20°-85°) and the mean supination for 60°(range, 30°-85°). The bony union time was 8-14 weeks (average 11 weeks) and the elbows were stable in flexion-extension and varus-valgus in all patients. The elbows maintained a concentric reduction of both the ulnotrochlear and the radiocapitellar articulation. Mild heterotopic ossification of the elbow occurred in 3 patients at 6 months after operation and mild degenerative change in 1 patient at 18 months after operation. The Broberg and Morrey elbow performance score was 82 points (range, 58-98 points). The results were excellent in 6 patients, good in 4, fair in 1 and poor in 1, with excellence rate of 83.3%. The results showed that the combined posterolateral and anteromedial approaches can facilitate the reduction and fixation of terrible triad of the elbow. Repair of radial head, coronoid, medial and lateral collateral ligaments can sufficiently restore the elbow stability, allow early postoperative motion and enhance the functional recovery.

## Introduction

Dislocation of the elbow combined with fractures of the radial head and the ulnar coronoid process is known as the terrible triad injury of the elbow. Treatment of this kind of injury is difficult. The traditional non-operative methods have very poor results because of recurrent instability and long-term fixation-induced stiffness. At present, many domestic and foreign scholars advocate operative treatment [1–21] to restore the concentric structure and stability of the elbow as well as allow the pain-free mobility of the elbow within the scope of functional activities. However, the surgical approach still remained unclear. Rodriguez-Martin et al [22] reported the results of 137 elbow triad injuries from five studies and an average of 31 months of follow-up showed that posterolateral and anteromedial approaches were effective in majority of patients, with Mayo elbow performance score of 85.6 points and Broberg-Morrey score of 85 points. Forthman et al [5] reviewed 34 patients and a mean follow-up of 32 months showed good to excellent results in 77% of patients according to the Broberg-Morrey score. Egol et al [23] reported 29 patients with a minimum of 1-year follow-up, which showed satisfactory results with mean Mayo elbow performance score of 81 points (range, 45–100 points) and mean Broberg-Morrey score of 77 points (range, 33–100 points). In this study, the operation methods and clinical results of posterolateral and anteromedial approaches for terrible triad injury of the elbow were analyzed in 12 patients admitted from January 2010 to June 2012 to discuss elbow stability, early postoperative motion and functional recovery of the elbow.

## Data and Methods

### General data

There involved 8 males and 4 females aged 22–55 years (average 33.6 years). The mechanism of injury included falling in 9 patients and traffic accidents in 3 patients, with 5 patients with the left elbow injury and 7 with the right elbow injury, all of which were closed injuries. Preoperatively, the patients underwent CT three-dimensional imaging diagnosis. According to O’Driscoll classification for fractures of the ulnar coronoid process, all patients were with type Ⅰ fractures (tip fractures); according to Mason classification for fractures of the radial head, there were 3 patients with type Ⅰ (fissure or marginal sector fractures with displacement), 7 with type Ⅱ (marginal sector fractures with displacement) and 2 with type Ⅲ (comminuted fractures involving the whole head). The period from injury to the operation was 2–13 days (4.6 days on average). All the patients signed the informed consent which was supervised by the Institutional Ethics Committee of Central Hospital of Yiwu City.

### Operation Methods

After admission, the manual reduction was done in all patients and the elbow joint was fixed externally. Operation was conducted after general conditions of patients became stable and the swelling disappeared. In the case of brachial plexus nerve block (10 patients) or general anesthesia (2 patients), the injured elbow was placed on a visible operation table, with a tourniquet for the proximal end of the upper arm.

Posterolateral elbow approach: The posterolateral elbow Kocher incision should start from the lateral epicondyle of humerus and enter via the gap between anconeus and extensor carpi ulnaris. This kind of injury is always combined with injury in the part between lateral soft tissue and deep fascia. The most common type is the laceration of lateral joint capsule and ligament at the posterolateral surface of lateral epicondyle of humerus. As a result, the lateral epicondyle of humerus is featured by bareness. The gap of injury can serve as the passage to joint, and annular ligament can be incised horizontally and vertically on the radial head. During the process of exposure, the forearm should be pronated to avoid the injury of deep branch of the radial nerve. Small screw (8 patients) or small T-shaped steel plate (2 patients) were used for fixation of MasonⅠand Ⅱ fractures. For Mason Ⅲ fractures, replacement of the radial head should be conducted. During the operation, the lateral collateral ligaments of 12 patients were injured at different degrees. The anchors were used to repair the lateral collateral ligaments.

Anteromedial elbow approach: The anteromedial approach started from the place 1–3 cm away from the medial epicondyle of humerus, when the distal end extended to the middle wrist joint and then stretched to the place 5 cm away from the ulnar coronoid process. The incision is 6–8 cm long. The skin and subcutaneous tissues were incised bluntly to identify and protect the medial cutaneous nerve of the forearm and ligate the veins obstructing the exposure. The medial intermuscular septum must be found to be released towards the proximal end. After defining the position of the crest of the medial epicondyle, the anterior structure at the distal end of humerus was lifted under periosteum to separate the pronator teres and flexors of the forearm longitudinally along the muscle fiber and incise the joint capsule in front of elbow joint. The coronoid process appeared and articular surface of the coronoid process tended to appear obviously when the elbow joint was completely extended. Anatomical reduction of fracture was conducted in an open manner. Kirschner wires were used for temporary fixation. The distal end of humerus should be completely located at the trochlear notch of olecranon before reduction and fixation of the coronoid process. Metacarpal support mini-plate fixation was used for all patients. Nine patients suffered from medial collateral ligament injury, 8 patients from laceration of medial epicondyle of humerus and 1 patient from laceration of sublime tubercle of the ulna, for which the anchor was used. With repair of bone and ligament structure, the stability of the elbow joint should be examined by X-ray. Through effective function activity scope for flexion of 30°-130°, the consistency of ulnotrochlear articulation should be examined carefully. Slight inconsistency may lead to sagging of the rear side of the radial head, implying insufficient repair of lateral soft tissue, which may lead to unstable posterolateral rotation and another repair is required.

### Postoperative treatment

After operation, the elbow joint was fixed at flexion of 90°, with the forearm at the neutral position. The regular anti-inflammation, swelling and pain treatment as well as oral indomethacin (at dose of 25 mg, 3 times per day for three weeks) were done for prevention of myositis ossificans. The exercise stated 2 weeks after operation. The flexion exercise should be done at first till the flexion of the elbow joint up to 90° for rotation of the forearm. At the 6^th^ week, the maximum extension was limited to 30°. After 8 weeks, the activity was not restricted and the patients could return to work normally. According to the need of activity intensity, the patients could normally restore heavy physical labor after 3 months. Within 3 months, the X-ray films should be taken for reexamination to ensure the concentric reduction of both the ulnotrochlear and the radiocapitellar articulations during healing process.

### Evaluation of curative effect

Clinical evaluation: The evaluation of the results was based on the function scoring standards of Broberg and Morrey [3]: 95–100 points as excellent, 80–94 points as good, 60–79 points as fair and 0–59 points as poor. The average score is 82 points (58–98 points).

Radiology evaluation: The anteroposterior and lateral X-ray films of the elbow joint were used for evaluating fracture healing, looseness of internal fixation, breakage, osteoarthritis, heterotopic ossification, and other conditions.

## Results

All patients were followed-up for 12–27 months (average 15.5 months), which showed that 10 patients had no pain but 2 patients had mild pain, with no severe pain. At the last follow-up, the average flexion was for 125° (range, 90°-140°), the average extension loss for 20° (range, 0°-70°) and the average pronation for 66° (range, 20°-85°), while the average supination was 60° (range, 30°-85°). The elbows were stable in flexion-extension and varus-valgus in all patients.

At the final follow-up, all coronoid process fractures and radial head fractures were healed without malunion, with bony healing time of 8–14 weeks (average 11 weeks). The elbows of 12 patients maintained a concentric reduction of both the ulnotrochlear and the radiocapitellar articulations. Mild heterotopic ossification (Brooker level Ⅰ) of the elbow occurred in 3 patients at 6 months after operation, which was given no special treatment. Mild degenerative change of the elbow occurred in 1 patient at 18 months after operation but showed no aggravation after rehabilitation exercise, subsidence of the radial head implants or significant capitellar bone wear.

**Fig 1:**
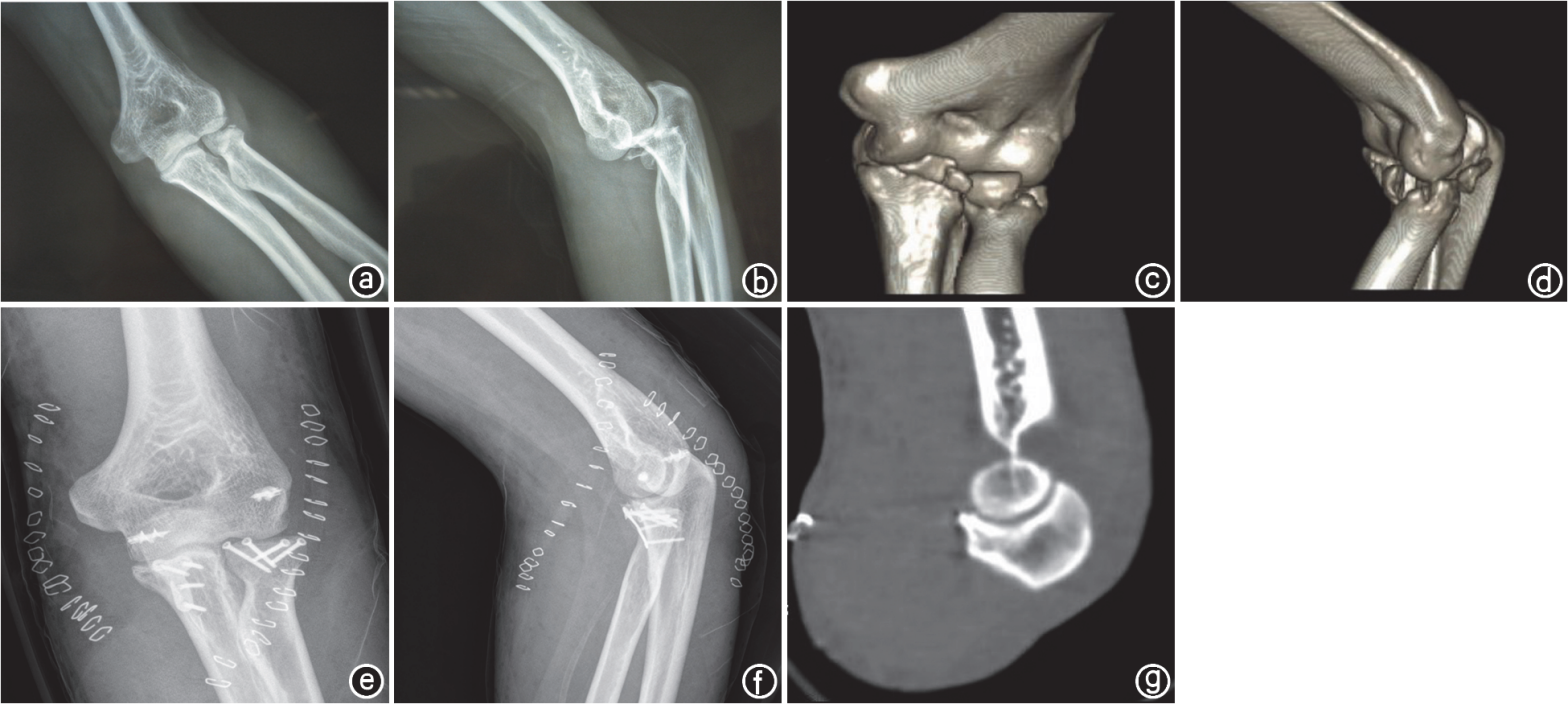
The male patient aged 47 years was admitted 2 days after left elbow injury induced by falls. a,b. Anteroposterior and lateral X-ray films before operation shows dislocation of left elbow joint, ulnar coronoid process fractures, and radial head fractures; c,d. Three-dimensional CT reconstruction before operation shows dislocation of left elbow joint, comminuted fractures of ulnar coronoid process, and comminuted fractures of radial head; e,f. Anteroposterior and lateral X-ray films were taken after fixation was conducted by applying medial and lateral elbow approaches; g. Three-dimensional CT reconstruction of vertical plane after operation indicates that elbow joint recovers with concentric reduction.

According to function scoring standards of Broberg and Morrey, the results were excellent in 6 patients, good in 4, fair in 1 and poor in 1, with excellence rate of 83.3%. See Fig 1.

## Discussion

There exists certain controversy over the selection of operative approach for the terrible triad injury of the elbow. Some scholars advocated the posterior longitudinal approach [4,5]. For terrible triad injury, a posterior skin incision has several advantages. One advantage is that it allows access to both the medial and lateral aspects of the elbow, and precludes the need for a second medial skin incision, when the posterior skin incision has a lower risk of injury to the cutaneous nerves compared with the medial and lateral skin incisions. Another advantage to the universal posterior approach is that it can also be used for elbow arthroplasty if required in the future. A disadvantage of a posterior incision is that the relatively large medial and lateral skin flaps increase the possibility of seromas and hematomas. Flap necrosis is also a potential complication, although it is rare in the setting of trauma [4]. Reichel et al [6] described a direct anterior approach to treat coronoid process fractures and an extra lateral approach to treat the radial head and LCL injury, with no complications in blood vessel nerve or wound, internal fixation failure, anterior heterotopic ossification or recurrent unstable elbow joint. At present, most scholars viewed that the lateral approach alone is enough for solving most problems [7,8]. However, some researchers thought that the combined medial and lateral elbow approaches could facilitate exploration and repair simultaneously and that treatment of each injured structure could improve the results [9]. In the study, the anteromedial and posteriorlateral elbow approaches were employed, in which the posterior-lateral approach was used for treating the radial head fracture and the lateral ligament injury while the anteromedial approach for treating the ulnar coronoid process fractures and the medial ligament injury. We think that the lateral approach alone cannot completely expose the ulnar coronoid process and operating space is limited. The fixation is more difficult when there exist comminuted fractures of ulnar coronoid process or fractures of medial articular surface of ulnar coronoid process. On the contrary, the anteromedial approach can provide sufficient exposure to facilitate the operating.

Ulnar coronoid process is the most important bony structure for stability of the front part of elbow joint and is the first factor for preventing backward dislocation and posterior-lateral subluxation of elbow joint. In addition, ulnar coronoid process serves as the attachment point for important soft tissues of elbow joint, including anterior joint capsule, bundle of medial collateral ligament, brachialis, etc. For fractures of the ulnar coronoid process, there still remains unclear at present. Thomas et al [10] indicated that type Ⅰ fractures are stable, with no necessity of operation. However, Papandrea et al [11] thought that type Ⅰ fractures of the ulnar coronoid process can obviously increase the instability of acute and chronic elbow joint as well as the risk of elbow joint degeneration after trauma, which is difficult to remedy. Ulnar coronoid process should be repaired as possible to facilitate the stability of joint [4].

Fixation of the ulnar coronoid process fractures includes screw fixation [12], anchor fixation [13], suture and stay rope repair through bone drilling [7,14] as well as fixation with steel plate and screw [15,16]. Most scholars suggest that the fractured pieces should be fixed by screw inserting from the posterior border of ulna into the coronoid tip. However, the patients with terrible triad injury of the elbow had smaller fractured pieces and screw fixation was difficult [12,13]. Such fixation may lead to insufficient holding force of the fractured pieces, which caused uneasy placing of the screws to the right position of the fractured pieces and finally resulted in failure of fixation. Due to small fractured pieces of ulnar coronoid process, most scholars apply suture Lasso repair through bone drilling [7,14] at present. Zeiders et al [7] applied the suture Lasso repair technology to repair the fractures of ulnar coronoid process in the terrible triad injury of the elbow in 32 patients. The average follow-up of 3 years showed activity scope of all elbows for 30°-130°, average extension loss for 12°(0°-20°) and average flexion loss for 14°(0°-20°). Three patients have reached complete activity scope. Garrigues et al [14] indicated that in the case of the terrible triad injury of the elbow, fixing fractures of ulnar coronoid process by applying suture Lasso repair technology can achieve higher stability and less complication than the screw or anchor fixation technology. Steinmann [15] thought that small steel plate can provide a support to resist posterior subluxation of the ulna. Fractures were firstly fixed by Kirschner wire or stabilized by large reduction forceps and then steel plates or screws were used for fixation. Doorenberg et al [16] used steel plates to firmly fix the fractures of ulnar coronoid process in 9 patients and thought that stable fixation of the coronoid process fracture can better recover functions of the elbow joints. The metacarpale mini-steel plate has gained satisfactory clinical results. The internal fixation of mini-steel plate can fix the fractured pieces in an integrated way, which can satisfy rigid internal fixation for fractures within the joints. After the operation, the joint function exercise can be conducted in the early stage. Besides, operating was simple and convenient during the operation, avoiding bone breakage at the time of fixation. Thickness of mini-steel plate is about 1 mm so that the plate can be shaped easily. After being inserted into the steel plate, the screws match well with the bone surface, with little interference on the joint activity and on the peripheral tissue especially activity of muscle tendon.

For the terrible triad injury of the elbow, the medial collateral ligament often suffers from laceration. Radial head is an important stable structure to resist elbow valgus and posterior dislocation of elbow joint. Ring et al [17] reported 11 patients with terrible triad injury of the elbow in 2002, including 4 patients treated with complete incision of the radial head and 3 patients with no repair of the lateral collateral ligament. The follow-up for average 7 years showed recurrent instability in 5 patients, including 4 patients with excision of the radial head. At the final follow-up, 7 patients suffered from joint osteoarthritis of ulnotrochlear articulation. They thought that radial head should be kept as possible. At present, scholars thought that during treatment of the terrible triad injury of the elbow, application of radial head excision alone should be prohibited. When the fractured pieces of the radial head are less than 3 pieces, open reduction and internal fixation should be applied as far as possible to maintain the integrity of the radial head. An appropriate reduction and stable fixation are the premise to allow activity in early stage and gain successful result[18]. Attention should be paid to the point that the tail end of screw should be completely embedded into annular articular surface of the radial head. When the fractured pieces of articular surface are more than 3 pieces, open reduction and internal fixation are difficult to make a success. Complex fractured pieces including compressed articular surface or small fractured pieces own few subchondral bones so that they are not suitable for internal fixation in any form. This increases the difficulty of operation fixation and the failure and malunion of fixation in early stage and later stage [19]. For those patients, replacement with metal radial head is a more reasonable choice.

The ligament structure should be evaluated after repair of the bone structure. Avulsion of the lateral collateral ligament off the lateral epicondyle of the humerus is commonly seen [20]. In this study, all patients suffered from avulsion from lateral epicondyle of humerus. Usually, the lateral collateral ligament is fixed to lateral epicondyle by using anchor or suture. Anchors are more expensive but the anchors enable the ligament to be fixed accurately to its anatomical position and facilitating the operating. Isotonic repair should be reached at the time of repair. Anchor is placed on rotation center of elbow joint and located at the center of capitellum curvature of the lateral epicondyle. Currently, there is a big controversy over whether repairing medial collateral ligament can increase the stability of the elbow joint. Some researches indicated that repairing medial collateral ligament will not increase the stability of the elbow joint. Forthman et al [5] conducted fracture fixation and lateral collateral ligament repair for 22 patients who suffered from terrible triad of the elbow, but they did not repair medial collateral ligament. The follow-up lasted for average 32 months, which showed excellence rate of functions for 77%, average activity degree of ulnotrochlear articulation for 117°, and average activity degree of forearm rotation for 137°. Therefore, they thought that only fixation and repair of the fractured pieces of the bones and lateral collateral ligament can achieve the stability of the elbow joint, when repair of the medial collateral ligament is useless. However, some researches [9,21] indicated that repair of medial collateral ligament and fixation of ulnar coronoid process and radial head fracture are of equal importance. Through biomechanics research, Beingessner et al [21] verified that medial collateral ligament injury had an obvious influence on activity and stability of the elbow joint. Therefore, if the elbow joint is still unstable after above-mentioned operation, it needs to judge whether there exists medial structure injury. If any, it should be repaired. Tulgar et al [9] suggested that repair of medial collateral ligament can reduce the occurrence of heterotopic ossification of ligament injury position and cubital tunnel syndrome. In this study, exploration of the medial collateral ligament was done when coronoid process fractures were repaired. Breakage of the medial collateral ligament happened in 9 patients. One patient suffered from avulsion off end point of the ulna while the rest suffered from avulsion off medial epicondyle. Anchors were used for repair in all patients. X-ray showed concentric reduction of all elbow joints, with no obvious instability during operation.

In short, treatment of the terrible triad injury of the elbow is difficult and attention should be paid to the injured bone and soft tissue structure. Combined posterior-lateral and anteromedial approaches can provide full exposure and easy and convenient operating and hence facilitate the reduction and fixation of the terrible triad injury of the elbow. Repair of radial head, coronoid process, medial and lateral collateral ligament can effectively recover the stability of elbow joint, allow early exercise postoperatively and facilitate recovery of the joint functions.

## References

[1] O’Driscoll SW, Jupiter JB, Cohen MS, Ring D, McKee MD. (2003) Difficult elbow fractures:pearls and pitfalls, Instr Course Lect 52:113–134. 12690844

[2] McKee MD, Jupiter JB. Trauma to the adult elbow//Browmer BD, Jupiter JB, Levine AM, eds. Skeletal Trauma[M]. 2nd ed. Harcourt Asia Saunders 2001:1455–1482.

[3] Broberg MA, Morrey BF. (1986) Results of delayed excision of the radial head after fracture. J Bone Joint Surg 68-A:669–674. 3722222

[4] Mathew PK, Athwal GS, King GJ. (2009) Terrible triad injury of the elbow: current concepts. J Am Acad Orthop Surg 17: 137–151. 19264707 10.5435/00124635-200903000-00003

[5] Forthman C, Henket M, Ring DC. (2007) Elbow dislocation with intra-articular fracture: the results of operative treatment without repair of the medial collateral ligament. J Hand Surg 32:1200–1209. 17923304 10.1016/j.jhsa.2007.06.019

[6] Reichel LM, Milam GS, Reitman CA. (2012) Anterior approach for operative fixation of coronoid fractures in complex elbow instability. Tech Hand Upper Extremity Surgery 16(2):98–104. 10.1097/BTH.0b013e31824e6a74 22627936

[7] Zeiders GJ, Patel MK. (2008) Management of unstable elbows following complex fracture-Dislocations the “Terrible Triad” injury. J Bone Joint Surg Am 90(4):75–84. 18984720 10.2106/JBJS.H.00893

[8] Kalicke T, Muhr G, Frangen TM. (2007) Dislocation of the elbow with fractures of the coronoid process and radial head. Arch Orthop Trauma Surg 127: 925–931. 17713772 10.1007/s00402-007-0424-6

[9] Toros T, Ozaksar K, Sügün TS, Kayalar M, Bal E, Ada S. (2012) The effect of medial side repair in terrible triad injury of the elbow. Acta Orthop Traumatol Turc, 46: 96–101. 10.3944/AOTT.2012.2632 22491433

[10] Thomas K, Gert M, Thomas MF. (2007) Dislocation of the elbow with fractures of the coronoid process and radial head. Arch Orthop Trauma Surg 127:925–931. 17713772 10.1007/s00402-007-0424-6

[11] Papandrea RF, Morrey BF, O’Driscoll SW. (2007) Reconstruction for persistent instability of the elbow after coronoid fracture-dislocation. J Shoulder Elbow Surg 16:68–77. 17067823 10.1016/j.jse.2006.03.011

[12] Seth D, Thomas F. (2013) Terrible triad of the elbow. Orthop Clin North Am 44:47–58. 10.1016/j.ocl.2012.08.006 23174325

[13] Pai V. (2009) Use of suture anchors for coronoid fractures in the terrible triad of the elbow. J Orthop Surg (Hong Kong) 17: 31–35. 19398790 10.1177/230949900901700108

[14] Garrigues GE, Wray WH 3rd, Lindenhovius AL, Ring DC, Ruch DS. (2011) Fixation of the coronoid process in elbow fracture-dislocations. J Bone Joint Surg Am 93: 1873–1881. 10.2106/JBJS.I.01673 22012524

[15] Steinmann SP. (2008) Coronoid process fracture. J Am Acad Orthop Surg 16: 519–529. 18768709

[16] Doornberg JN, de Jong IM, Lindenhovius AL, Ring D. (2007) Fracture of the anteromedial facet of the coronoid process. J Shoulder Elbow Surg 16: 667–670. 17512221 10.1016/j.jse.2007.03.013

[17] Ring D, Jupiter JB, Zilberfarb J. (2002) Posterior dislocation of the elbow with fractures of the radial head and coronoid. J Bone Joint Surg Am 84-A: 547–551. 11940613 10.2106/00004623-200204000-00006

[18] Kraig YB, Randeep SK. (2006) Radial head fractures-advanced techniques in surgical management and rehabilitation. J Hand Ther 19:114–135. 16713860 10.1197/j.jht.2006.02.011

[19] Ring D, Quintero J, Jupiter JB. (2002) Open reduction and internal fixation of fractures of the radial head. J Bone Joint Surg Am, 84-A:1811–1815. 12377912 10.2106/00004623-200210000-00011

[20] McKee MD, Schemitsch EH, Sala MJ, O'driscoll SW. (2003) The pathoanatomy of lateral ligamentous disruption in complex elbow instability. J Shoulder Elbow Surg 12:391–396. 12934037 10.1016/s1058-2746(03)00027-2

[21] Beingessner DM, Stacpoole RA, Dunning CE, Johnson JA, King GJ. (2007) The effect of suture fixation of type Ⅰ coronoid fractures on the kinematics and stability of the elbow with and without medial collateral ligament repair. J Shoulder Elbow Surg 16:213–217. 17399625 10.1016/j.jse.2006.06.015

[22] Rodriguez-Martin J, Pretell-Mazzini J, Andres-Esteban EM, Larrainzar-Garijo R. (2011) Outcomes after terrible triads of the elbow treated with the current surgical protocols. A review. Int Orthop 35: 851–860. 10.1007/s00264-010-1024-6 20449590 PMC3103950

[23] Egol KA, Immerman I, Paksima N, Tejwani N, Koval KJ. (2007) Fracture-dislocation of the elbow functional outcome following treatment with a standardized protocol. Bull NYU Hosp Jt Dis 65:263–270. 18081545

